# Herpes Simplex Virus Encephalitis in Hamadan, Iran

**Published:** 2013-09

**Authors:** Masoud Sabouri Ghannad, Ghasem Solgi, Sayed Hamid Hashemi, Javad Zebarjady-Bagherpour, Ali Hemmatzadeh, Mehrdad Hajilooi

**Affiliations:** 1 Departments of Microbiology, Faculty of Medicine, Hamadan University of Medical Sciences, Hamadan; 2 Departments of Immunology and Faculty of Medicine, Hamadan University of Medical Sciences, Hamadan; 3 Departments of Infectious Diseases, Faculty of Medicine, Hamadan University of Medical Sciences, Hamadan; 4 Research Center for Molecular Medicine, Hamadan University of Medical Sciences, Hamadan, Iran

**Keywords:** Herpes Simplex Virus, Encephalitis, mortality, Cerebrospinal Fluid

## Abstract

**Background and Objectives:**

Encephalitis can cause a severe public health problem. The main aim of this research was to evaluate the medical laboratory results of patients with *Herpes Simplex Virus* (HSV) encephalitis.

**Materials and Methods:**

Diagnosis of encephalitis for these patients was firstly based on a clinical profile for Herpes Simplex Encephalitis (HSE), plus either a detected HSV1&2-DNA by PCR in CSF or brain neuro-imaging results.

**Results:**

Molecular testing on CSF showed that 15 patients (15%) had HSV infection, 5 patients (5%) had *Varicella Zoster Virus* (VZV) and one case was positive for *Human Immunodeficiency Virus* (HIV)-RNA in CSF. The cause of encephalitis in 79 out of 100 patients (79%) was unknown. The comparison of CSF analysis in HSV positives and negatives showed a significant increase of glucose and protein levels in HSV positives than negatives. The mortality rate was 46.6% (7/15) in patients with HSV encephalitis compared to 11.4% (10/85) in non-HSV encephalitis (P *=* 0.003).

**Conclusions:**

In the current study, 15% of cases were diagnosed as having HSV.

## INTRODUCTION

Encephalitis is an inflammatory process of the brain parenchyma which results in brain dysfunction and must be noted seriously in a patient who manifests with headache, fever, and altered level of consciousness. Even mild forms of any neurologic sign including meningismus, headache, and altered mental status should intrigue an assessment for any potential CNS infection. The etiology of encephalitis can be separated into two major causes (1); infection-related encephalitis by a direct effect of pathogenic infectious agents and/or also autoimmune-mediated encephalitis possibly induced by vaccination or a recent viral infection (1). *Herpes simplex virus* (HSV1&2) and *Varicella-Zoster Virus* (VZV) are considered as the most common etiology of acute infectious encephalitis (1). Herpes simplex encephalitis (HSE) is reported as the most reason of infrequent necrotizing encephalitis in adults (2). Encephalitis is correlated with considerable morbidity and mortality, but its cause remains to be fully understood. Multiple strategies have been employed during the past decade to recover the effects of HSV infection. Nevertheless, mortality and morbidity rates from HSE and neonatal HSV disease remain inappropriately high (3).

To the best of our knowledge, there are scarce data available from the causes of encephalitis in Iran and this is the first research which evaluates the prevalence of HSV encephalitis which is informative in Hamedan, a western province of country. Consideration of factors responsible for encephalitis can be essential in the evaluation of the incidence rate of severe complications (4).

## MATERIALS AND METHODS

### Sample collection

This is a retrospective study in 100 recorded questionnaires of patients who hospitalized due to encephalitis in Sina University Hospital in Hamadan, Iran from April 2004-to March 2012 over an 8-year period. Our study was approved by the Ethics Committee of Hamadan University of Medical Sciences in accordance with the tenets of the Helsinki Declaration and the national ethical guideline for medical research.

CSF sampling has been performed once following patients’ hospitalization. Diagnosis of encephalitis for these patients was firstly based on a clinical profile for HSE, plus either a detected HSV1&2-DNA by PCR in CSF or brain neuro-imaging results. All 100 CSF samples were tested by PCR to look for HSV. Molecular detection of HSV-DNA and also *Mycobacterium tuberculosis* (TB) in CSF samples was performed for all patients by commercial PCR kits (DNA-Technology Co., Russia). Also, some HSV negative cases were tested for *Human Immunodeficiency Virus* (HIV) infection and Varicella Zoster Virus (VZV) by conventional PCR method. The demographic data and laboratory findings including age, gender, clinical symptoms, CSF parameters, hematological tests and the mortality rate were analyzed in relation to HSV infection as an etiological factor in this study. The demographic data and laboratory findings were assessed by software SPSS 16.

### Statistical analysis

The results were analyzed using Prism 5.01 (Graph pad Software, San Diego, CA, USA). Baseline characteristics were summarized as means and proportions of selected variables. The mean values of quantitative variables between groups were compared using an unpaired t-test for data distributed normally and a Mann–Whitney test for non-normal data. Categorical variables were analyzed by Chi-Square test and fisher exact test. All tests were considered significant if *p values* were less than 0.05.

## RESULTS

Clinical diagnosis of encephalitis was confirmed in 100 patients. Results of CSF bacterial culture of all the samples were negative so PCR test was performed for HSV in all of the cases. PCR tests were negative in 79 samples.

Analysis of the demographic data and clinical manifestations as well as laboratory findings was performed in all patients with the signs and symptoms of encephalitis. The results of *Mycobacterium tuberculosis* (TB) PCR in CSF samples were negative for all patients.

Molecular testing on CSF showed that 15 patients (15%) were diagnosed as having HSV infection, 5 patients (5%) had VZV and one case was positive for HIV-RNA in CSF (dead case). However, all patients were negative for TB in CSF by molecular testing.

The cause/s of encephalitis in 79 out of 100 patients (79%) was unknown. The median age of hospitalized patients with encephalitis was 28 years old. Out of 100 patients, 56 cases were female and 44 were male.

More than half of patients under the study (55.3%) presented fever and 40.4% of the patients had headache. Patients with decreased level of consciousness and seizure symptoms were as 34.04% and 31.9%. Vomiting, nausea, altered mental status, and aphasia were reported in 23.4%, 19.1%, 10.6%, and 6.4% of the patients respectively (Table 1). Demographics of HSE cases are shown in Table 2. Out of 15 cases with herpetic encephalitis, deaths were reported in 7 cases between 3 to 10 days after hospitalization (Table 2).

**Table 1:** Demographics of available data in the patients with herpes encephalitis and non-herpes encephalitis.

*Variables*	Patients (N = 100)
Age in Years (Median)	1.5-83 (28)
Gender (F /M)	56 /44
**Etiology**	
Herpetic Encephalitis	15 (15.0%)
VZV	5 (5%)
HIV	1 (1%)
Unknown	79 (79%)
**Clinical Symptoms**	
Fever	55.3%
Headache	40.4%
Decreased level of consciousness	34.04%
Seizure	31.9%
Vomiting	23.4%
Nausea	19.1%
Altered mental status	10.6%
Aphasia	6.4%
**CSF parameters (median)**	
WBC*	0-1500 (40)
PMN*	3-97 (37.5)
Lym*	3-97 (65)
Protien (mg/dl)	0-470 (50)
Glucose (mg/dl)	12.5-187 (64.5)
**Hematologic**	
WBC	4300-21400 (9100)
PMN	36-97 (80)
Lym	7-60 (18)

* Number of cells per microliter of CSF sample. VZV: Varicella zoster virus, HIV: Human Immunodeficiency Virus, WBC: White blood cells, PMN: Polymorphonuclear cells, Lym: Lymphocyte.

**Table 2:** Demographic data and laboratory findings of the 15 patients with herpes simplex virus encephalitis.

Patient's No.	Age/gender	Symptoms	CSF WBC (Cells/ml)	CSF Protein (mg/dl)	CSF Glucose(mg/dl)	Outcome*(Days after hospitalization)
1	33/M	Fever, DLC, Headache	0	190	54	Dead (day 9)
2	45/M	Fever, Seizure, DLC, Headache	250	470	113	Dead (day 4)
3	13/F	Fever, Conjunctivitis	10	12	45	Dead (day 7)
4	22/F	DLC, Hemi paresis, nausea, Aphasia	10	360	102	Dead (day 10)
5	16/F	Seizure, DLC	40	180	165	Dead (day 8)
6	22/F	Headache, vomiting, nausea, strabismus	10	360	102	Dead (day 4)
7	83/F	Fever, DLC	0	30	132	Dead (day 3)
8	27/F	Fever, Seizure, DLC	10	20	78	Discharged (day 15)
9	40/M	Headache, vomiting, nausea, aphasia	190	50	60	Discharged (day 23)
10	15/F	Fever, Seizure	125	34	71	Discharged (day 20)
11	56/F	Seizure, vomiting, diarrhea, sleepy	130	120	103	Discharged (day 44)
12	83/F	Fever,	100	108	187	NR
13	34/M	Seizure, DLC, weakness, Fatigue	50	60	58	Discharged (day 26)
14	66/F	Fever, DLC, AMS,	70	86	47	Discharged (day 27)
15	24/M	Headache, vomiting, Fever, sed	1100	350	33	NR

AMS: Altered mental status, DLC: Decreased level of consciousness, NR: Not reported

* Clinical events after hospitalization (death or discharged)

The outcome of 2 patients remained unreported. Discharge of patients occurred between 15-44 days following hospitalization and treatment (Table 2). The comparison of HSV positives and negatives showed that the mean age of HSV encephalitis and non-HSV encephalitis were 38.40 ± 5.67 and 28.7 ± 4.04 respectively (P value = 0.17).

The comparison of CSF analysis in HSV positives and negatives showed a significant increase of glucose (P value = 0.01) and protein levels (P value = 0.002) in HSV positives than negatives (Table 3).

**Table 3:** Comparison of HSV positives and negatives.

Parameters	HSV- encephalitis (N = 15)	Non-HSV encephalitis(N = 85)	P values
Age (Mean±SD)	38.4±5.67	28.7±4.04	0.17^a^
No of dead cases	7/15	10/85	0.003^b^
**CSF analysis**			
WBC (Mean±SD)	131.3±67.23	156.2±46.7	0.75
PMN (Mean±SD)	46.3±8.7	39.2±6.6	0.56
Lym (Mean±SD)	53.7±8.7	70.07±5.9	0.06
Protein (mg/dl)	157.3±36.45	58.2±9.44	0.002
Glucose (mg/dl)	90.2±11.7	63.9±4.05	0.01
**Hematologic**			
WBC (Mean±SD)	8813±752.8	10210±742	0.29
PMN (Mean±SD)	80.63±2.38	75.24±2.68	0.18
Lym (Mean±SD)	20.38±2.69	21.31±2.47	0.81

a Unpaired t- test

b Two-tailed P values by fisher exact test.

A mild leukocytosis and lymphocytosis were also observed in patients with non-HSV encephalitis. The comparison of hematologic tests in patients showed a mild increase in the number of WBC in peripheral blood but with insignificant difference. PMN and lymphocytes in 2 groups also showed no significant difference (Table 3).

The number of dead cases in patients with HSV encephalitis were 7 out of 15 versus 10 out of 85 non-HSV encephalitis cases (P = *0.003*). In other words, mortality rate was 46.6% in patients with HSV encephalitis and 11.7% in non-HSV encephalitis.

## DISCUSSION

Encephalitis can cause a severe public health problem. A rapid and accurate diagnosis is vital for early detection and also efficient treatment which may improve the results of such infections. However, a study in France indicated that management of acyclovir in encephalitis patients was successful and the rate of mortality due to HSV infection was low in spite of admission of 49% of patients to intensive-care units (5).

We reviewed hospitalizations from HSV encephalitis in Hamadan, Iran. Results of CSF bacterial culture of all the samples were negative. So, PCR test was performed for HSV types 1 & 2 and TB in all of the cases. The reason for CSF negative bacterial culture may likely to be due to initiation of antibiotic therapy in all patients which makes the cause of non herpetic encephalitis ambiguous. Nevertheless, this issue remains to be clarified. Because of the prevalence of TB in this region, all the results were tested for TB as well. It was more interesting that all the samples were negative for TB.

Age was a non significant factor in our research in the patients with HSV encephalitis and non HSV encephalitis. This observation is in line with a similar experience that confirms the age factor as an independent prognostic factor of the disease (2).

Out of total 100 patients included in the current study, 15 (15%) cases were diagnosed as having HSV. This is in agreement with a report from England which shows the incidence of HSV as 19% in patients with encephalitis (6). However, a report from France shows that the most frequent etiologic agent of encephalitis was HSV (7). In comparison to our results, a previous report from Shiraz, a city in the south of Iran, showed the positive detection rate of encephalitis as 9.3% (8). Nevertheless, a report from Babol, a northern city in Iran, reported HSV positive in a high rate (34%) of patients with encephalitis (9). A research in Canada reports HSV as a causative factor of encephalitis in 0.3% of children with febrile seizures (10). Also, in Taiwan HSV is still considered as the main viral source of encephalitis (11). However, a study from Spain indicated that HSV was positive in 92% of patients hospitalized with encephalitis (2). The high rate of HSV infection in that research may be due to high quality of sampling and laboratory diagnosis methods.

The main proportion of patients in the current study were the rural dwellers. There is no complete document on the information requirements of rural inhabitants. Delays for hospitalization and diagnosis, unavailability of antivirals, inappropriate sampling for PCR test may be some factors which interfere with on time treatment of the patients. Future efforts should include giving access to information and knowledge by non-literates in the rural inhabitants.

We observed five cases (5.0%) with VZV as an etiological factor for encephalitis which is similar to a report from England with 5% VZV as the known etiology of encephalitis (6). Accordingly, Mamani et al., reported that 78.5% of pregnant women in Hamadan had protective levels of IgG against VZV that confirms the sensitivity of a significant section of this group to the preliminary VZV infection (12). Also, the results of that study show the incidence of VZV in this part of Iran which justifies our result as well.

The etiology of encephalitis in 79 out of 100 hospitalized patients (79%) was unknown through the clinical diagnosis systems and routine laboratory tests, leading in under diagnosis of a large proportion of encephalitis in Hamadan, Iran. In the current research, no testing was performed to look for other important etiologies including enteroviruses and arboviruses. Also, there was no data available to show which arboviruses are endemic in the area. These were limitations of our study. Nevertheless, this is in agreement with the literature which shows the cause of over 75% of hospitalizations with encephalitis has not been reported in England (13). However, in the current study the reason of unknown etiology might be explained by the fact that if the CSF specimens is provided too early (post 24-48 hrs of infection) or too late (after 10-14 days of infection), then false negative PCR may be occurred (9).

Another report also confirms that if CSF samples are collected within a few days following the beginning of clinical manifestations while white blood cell count (WBC) and CSF protein are not extremely increased, the PCR test in HSE patients may change to negative (8). These remain controversial which needs to be elucidated.

In our study, no significant correlation was found between biochemical parameters and also hematological findings in positive PCR cases except the level of glucose and protein in the CSF. Analysis of CSF in HSV positives and negatives showed a significant increase of glucose (P value = 0.01) and protein levels (P value = 0.002) in HSV positive versus negative patients. In comparison to the current study, a research in Thailand showed the decreased contents of CSF glucose and increased levels of CSF protein in both HSE and also in a control group with other viral encephalitis (14).

CSF pleocytosis was reported in our research. This is in consistent with the CSF leukocytosis with lymphocyte predominate which was also reported in both HSE and also in other viral encephalitis (14). Also, a mild leukocytosis and lymphocytosis was seen in patients with non-HSV encephalitis. The comparison of hematologic tests in patients also indicated a mild increase in the number of WBC in peripheral blood but with insignificant difference. PMN and lymphocytes in both HSE encephalitis and non encephalitis patients also showed no significant difference (Table 3).

We found that, fever was the most common symptom at the time of hospitalization. So more than half of patients under the study (55.3%) presented fever and 40.4% of the patients had headache. Other symptoms in patients were decreased level of consciousness and seizure as 34.04% and 31.9%. Vomiting, nausea, altered mental status, and also aphasia were reported in 23.4%, 19.1%, 10.6%, and 6.4% of the patients under study respectively.

We suppose that HIV has been implicated in causing the death in 1 out of 100 patients with encephalitis. In this patient HSV and TB tests were negative. This is in agreement with the reports that confirm HIV encephalopathy (15, 16). Regarding to the majority of patients with unknown encephalitis in our study, this result may suggest HIV as a pathogen for this group of people. Thus, we recommend performing PCR as a choice which is useful in identifying the patients with non-herpetic encephalitis.

The mortality rate was 46.6% in patients with HSV encephalitis compared to 11.7% in non-HSV encephalitis (P value = 0.003, Table 3). Therefore, the sanitation authorities may highlight the importance of prioritization in accurate diagnosis, early detection of disease, appropriate treatment and follow-up of the infected patients with HSV in this part of country (17). It is also suggested that in suspected patients with the preliminary negative HSV DNA in CSF, the PCR test be repeated within 48-72 hours to reconfirm the results of diagnosis.

The unknown encephalitis remains to be investigated in Hamadan, Iran. The non detectable infectious agents of encephalitis in this part of country and the high rate of disease with no known etiology may highlight the appearance of novel pathogens. Ongoing attempts may weave the other causative pathogens of patients with encephalitis. Further investigation is needed to analyze this observation in detail and to determine the function of different microbial agents in CNS infections.

In conclusion, attention should be drawn to herpetic encephalitis in the patients admitted with an unexpected alteration in level of consciousness in a formerly healthy patient with fever and previous viral infection. Our research provides a fascinating new insight into the nature of the clinical and etiologic patterns of herpetic encephalitis in this geographic region. The picture that has been emerged from the current study indicates the high rate of encephalitis from unknown pathogens in Hamadan which in turn underlies the specific features of monitoring the morbidity and mortality resulted from herpetic infection and detecting the possible emerging infectious diseases. This justifies the need for expanding our information about HSE pathogenesis to other potential effective antiviral and anti inflammatory treatment targets which are more prone to progress.
